# NLC-Based Rifampicin Delivery System: Development and Characterization for Improved Drug Performance Against *Staphylococcus aureus*

**DOI:** 10.3390/pharmaceutics17060799

**Published:** 2025-06-19

**Authors:** Javiera Carrasco-Rojas, Felipe I. Sandoval, Christina M. A. P. Schuh, Carlos F. Lagos, Javier O. Morales, Francisco Arriagada, Andrea C. Ortiz

**Affiliations:** 1 Departamento de Ciencias y Tecnología Farmacéutica, Facultad de Ciencias Químicas y Farmacéuticas, Universidad de Chile, Santiago 8380494, Chile; javiera.carrasco@uchile.cl (J.C.-R.);; 2 Centro de Medicina Regenerativa, Facultad de Medicina, Clínica Alemana-Universidad del Desarrollo, Santiago 7550000, Chile; 3 Escuela de Química y Farmacia, Facultad de Ciencias, Universidad San Sebastián, Campus Los Leones, Lota 2465, Providencia, Santiago 7510157, Chile; 4 Centro Basal Ciencia & Vida, Fundación Ciencia & Vida, Av. del Valle Norte 725, Huechuraba 8580702, Chile; 5 Centro de Nuevos Fármacos para Hipertensión e Insuficiencia Cardíaca, CENDHY, Santiago 8380494, Chile; 6 Centro Avanzado de Enfermedades Crónicas, ACCDiS, Santiago 8380494, Chile

**Keywords:** nanostructured lipid carrier, antibiotic, rifampicin, drug release, *Staphylococcus aureus*, HepG2 cell

## Abstract

**Background/Objectives**: Rifampicin is a typical antibiotic used for the treatment of *Staphylococcus aureus* (*S. aureus*) infections; however, its clinical utility is limited by poor aqueous solubility, chemical instability, and increasing bacterial resistance. Nanostructured lipid carriers (NLCs) offer a promising strategy to improve drug solubility, stability, and antimicrobial performance. **Methods**: In this study, rifampicin-loaded NLC (NLC-RIF) was developed using a hot homogenization with a low energy method and characterized in terms of particle size, polydispersity index, zeta potential, encapsulation efficiency, colloidal stability, and drug loading. **Results:** In vitro release studies under sink conditions demonstrated a biphasic release pattern, best described by the Korsmeyer–Peppas model, suggesting a combination of diffusion and matrix erosion mechanisms. Antimicrobial activity against *S. aureus* revealed a substantial increase in potency for NLC-RIF, with an IC_50_ of 0.46 ng/mL, approximately threefold lower than that of free rifampicin. Cytotoxicity assays in HepG2 cells confirmed over 90% cell viability across all tested concentrations. **Conclusions**: These findings highlight the potential of NLC-RIF as a biocompatible and effective nanocarrier system for enhancing rifampicin delivery and antibacterial activity.

## 1. Introduction

Global concern about multidrug resistance to antimicrobials has increased in recent years. The World Health Organization has emphasized the need to develop new strategies to combat antimicrobial resistance [1]. Conventional antibiotics still suffer from limitations such as low bioavailability, rapid clearance, and poor penetration into infection sites, often aggravated by biofilm formation. These shortcomings contribute to therapeutic failures, rising resistance levels, and patient discontinuation of treatment due to lack of efficacy. To overcome these barriers, nanoscale delivery systems can be employed to encapsulate, target, and release drugs at specific sites, thereby improving treatment efficacy, reducing dosage, and enhancing patient adherence [2,3,4].

In this context, two main categories of nanoantibiotics have been described [5]. The first corresponds to nanosystems that have antibacterial activity in themselves, such as silver, zinc, and gold nanosystems. The second group is nanoparticles capable of conjugating antibiotics on the surface or incorporating them into the matrix of the nanosystem. Nanotechnology is an appropriate strategy to circumvent bacterial resistance mechanisms because it circumvents the mechanisms that bacteria have developed for drugs. Additionally, improves key physicochemical and biopharmaceutical properties of drugs, such as water solubility and half-life [2,6]. Furthermore, strategies can be developed to design modified releases that could reduce the frequency and dose of administration [7], and may enable synergistic effects by co-delivering multiple antibiotics in a single formulation [8,9].

Among the various nanocarrier types, solid lipid nanoparticles (SLN) and nanostructured lipid carriers (NLCs) are particularly attractive due to their low toxicity, high stability, biocompatibility, biodegradability, and ability to encapsulate both hydrophilic and lipophilic drugs [10]. Moreover, their manufacturing is cost-effective and readily scalable [11].

Specifically, NLCs mitigate burst release during storage by their reduced recrystallization rate. This arises from a less-ordered lipid matrix formed by mixing solid and liquid lipids, which creates additional spaces for drug accommodation [12]. Accordingly, NLCs have emerged as versatile platforms to optimize the delivery of various therapies, including antibiotics [13]. Moreover, these nanoparticles can be fabricated using a combination of lipids and polymer-lipid conjugates, including polyethylene glycols (PEGs) of varying lengths and end-moieties or functionalized with different polymers [14,15]. This approach enables multiple functionalizations with various agents to direct the nanosystem to specific targets [16]. Therefore, they combine excellent biocompatibility and long-term drug retention with surface properties amenable to targeting ligands, while their nanoscale dimensions facilitate enhanced interactions with bacterial membranes [17,18].

Rifampicin is a hydrophobic, zwitterionic antibiotic characterized by both acidic and basic functional groups, pKa 1.7 and 7.9, respectively. It is regarded as one of the most potent bactericidal agents and is widely used to treat infections caused by surface-adherent microorganisms [19]. Its therapeutic efficacy is attributed to its broad-spectrum antimicrobial activity, which includes most Gram-positive bacteria and select Gram-negative species. However, rifampicin is not exempt from pharmacokinetic drawbacks, including variable solubility and instability in different conditions of pH, which may limit their efficacy against resistant strains and intracellular pathogens [20]. Rifampicin remains a cornerstone of tuberculosis therapy; in 2012, an estimated 450,000 cases of multidrug-resistant tuberculosis were reported globally, 10% of which were extensively drug-resistant [21]. Resistance to rifampicin in *Staphylococcus aureus* remains relatively low (~6.2%), yet vigilance is warranted given its potential to increase over time [22]. Therefore, strategies to reduce the emergence of antimicrobial resistance are urgently needed.

The high lipophilicity of rifampicin (LogP 2.83) favors its encapsulation within lipid matrices [10], while its ionizable groups support the controlled release into aqueous environments. Although rifampicin-loaded nanosystems have been evaluated against *Mycobacterium tuberculosis* [23], *Escherichia coli* [24], *Pseudomonas aeruginosa* [25], *Klebsiella pneumoniae* [26], and *Staphylococcus aureus* [27]. To our knowledge, no study has yet reported rifampicin encapsulation in NLCs specifically against *Staphylococcus aureus* [28,29,30].

In this study, we developed nanostructured lipid carriers (NLCs) for the encapsulation and controlled release of rifampicin, with a primary focus on evaluating their biological performance. We conducted a comprehensive biological assessment, including antimicrobial activity against *Staphylococcus aureus* and cytotoxicity using a hepatic cell model to investigate biocompatibility. Additionally, physicochemical characterization and colloidal stability of the nanosystems, along with in vitro release kinetics, were evaluated to support the biological findings. Our results contribute to the growing evidence supporting NLCs as promising platforms for advanced antibiotic delivery, offering a potential route to enhance drug performance and address the ongoing challenge of bacterial resistance.

## 2. Materials and Methods

### 2.1. Materials

Gelucire^®^ 44/14 was donated by Gattefosse. Miglyol^®^ 812, Tween^®^ 80, rifampicin (≥95% HPLC), dimethyl sulfoxide (DMSO, Molecular Biology), 3-(4,5-dimethylthiazol-2-yl)-2,5-diphenyltetrazolium (MTT, ≥97.5%), Amicon^®^ Ultra 10 KDa, and triple sugar iron medium (TSI) were purchased from Merck (Merck KGaA, Darmstadt, Germany). SnakeSkinTM Dialysis Tubing of cellulose membrane with a MWCO 10 kDa was purchased from Thermo Fisher Scientific (Thermo Fisher Scientific, Carlsbad, CA, USA). Acetic acid (HPLC grade) and acetonitrile (HPLC grade) were purchased from PanReac AppliChem ITW Reagents (Darmstadt, Germany). Dulbecco’s Modified Eagle Medium (DMEM high-glucose), phosphate-buffered saline (PBS, without calcium and magnesium), fetal bovine serum (FBS), and penicillin/streptomycin solution were obtained from Cytiva. Trypsin-EDTA solution (0.25%) was purchased from Gibco. Ultra-pure water (18.2 MΩcm) was produced using a Simplicity System from Millipore. All materials were used as received without any further purification.

### 2.2. Preparation of NLCs

The fabrication process was described in a previous report [31]. The NLC is composed of Gelucire^®^ 44/14, Miglyol^®^ 812, Tween^®^ 80, and water. Briefly, for a 100% formulation, the lipid phase consisted of 4% *w*/*w* Gelucire^®^ 44/14, 1% *w*/*w* Miglyol^®^ 812, and 2% *w*/*w* Tween^®^ 80, which were mixed and heated to 85 °C. Meanwhile, the aqueous phase, comprising 93% *w*/*w* water heated to 85 °C, was slowly added to the lipid phase under constant stirring at 400 rpm. Then the mixture was then cooled rapidly at 4 °C for at least 30 min without stirring. To prepare rifampicin-loaded NLC, rifampicin (50 mg) was incorporated into the lipid phase, and the process continued as described. We previously determined the optimal amount of rifampicin loaded into the nanocarrier. The resulting formulation was designated NLC-RIF.

### 2.3. Incorporation Efficiency and Drug Loading

To quantify the different drugs incorporated in NLC, the formulations were ultracentrifuged in 10,000 Da MWCO Amicon^®^ Ultra for 15 min at 6900 rcf. Then, the filtered section was separated and quantified through HPLC.

The rifampicin method was adapted from Goutal and collaborators [32]. Chromatographic analysis was performed using a Shimadzu Prominence HPLC system (Kyoto, Japan). The separation was achieved using an Inertsil ODS-3 C18 column (GL Science Inc., Tokyo, Japan) (4.6 mm × 250 mm, 5 μm). The mobile phase was composed of water/acetic acid 0.01% (phase A) and acetonitrile/acetic acid 0.01% (phase B). The gradient elution mode considers a change of phase B to phase A increased linearly from 30% to 60% from 0 to 10 min. The flow was set at 1.2 mL/min while the injection volume was 50 μL. Rifampin was detected at 7.3 min during a total run time of 13 min at a wavelength of 335 nm.

The drug loading and incorporation efficiency percentages were determined according to the following Equations (1) and (2), respectively:

(1) $$\%\;\mathrm{Incorporation}\;\mathrm{efficiency}=\frac{{Rifampicin}_{total}-{Rifampicin}_{free}}{{Rifampicin}_{total}}*100$$

(2) $$\%\;\mathrm{Drug}\;\mathrm{loading}=\frac{{Rifampicin}_{total}-{Rifampicin}_{free}}{\mathrm{Solid}\;\mathrm{lipid}\;+({Rifampicin}_{total}-{Rifampicin}_{free)}}*100$$

where Rifampicin_total_ and Rifampicin_free_ are initial drug amounts, incorporated into the NLC-RIF preparation. The unloaded drug amount corresponds to the evaluated by the corresponding HPLC method.

### 2.4. Physicochemical Characterization

To determine hydrodynamic diameter (HD), polydispersity index (PdI), and zeta potential (ZP), a Malvern Zetasizer Nano ZS equipment (Malvern Panalytical Ltd., Malvern, UK) was used. HD and PdI were measured by dynamic light scattering (DLS) and ZP was determined by Doppler laser microelectrophoresis. Samples were measured in an aqueous medium with a dilution of 1:10 and each determination was measured three times corresponding to the average of eleven determinations. The condition of the medium presents a viscosity of 0.08872 cP, refractive index of 1.330, and sample refractive index of 1.333 with a wavelength of 633 nm with a detection angle of 173° and equilibration time of 120 s. Disposable cuvettes (model DTS0012) and disposable folded capillary cells (model DTS1060) were used for HD and ZP measurements, respectively.

On the other hand, to determine the formulation concentration, a nanoparticle tracking analysis (NTA) NanoSight NS300 (Malvern Panalytical, Malvern, UK) was used. The diluted samples were injected with sterile syringes into the sample chamber equipped with a 532 nm diode laser (green). All samples were measured in a single shutter and gain mode for 90 s with manual shutter, gain, brightness, and threshold adjustments at room temperature.

### 2.5. Colloidal Stability Assessment

The colloidal stability of all types of nanoparticles was determined over 1, 2, 3, 4, 7, and 8 weeks at 4 °C. In each case, HD, PdI, and ZP were registered.

### 2.6. Morphology of Nanoparticles

To visualize the NLC and NLC-RIF morphology, transmission electron microscopy was performed. Briefly, NLC and NLC-RIF were diluted 100 times with water, deposited on film-coated copper grids, stained with 1% phosphotungstic acid for 2 min, washed with water, and dried for 48 h at room temperature. The samples were evaluated on an Inspect F50 Scanning Transmission Electron Microscope (FEI, Hillsboro, OR, USA).

### 2.7. Drug Release Studies

Release of rifampicin-loaded NLC and free rifampicin was measured using the dialysis bag method. SnakeSkin ^TM^ dialysis bag of 10 kDa MWCO was embedded in 10 mL of PBS (pH 7.4) containing 0.1% of Tween^®^ 80 at 37 °C. Samples (1 mL) were withdrawn at selected time intervals for 72 h and replaced with 1 mL of fresh medium. The amount of release drugs was quantified in triplicate by HPLC methods described previously. Once the data were obtained, kinetic models were used to fit the release profile of studies and the coefficient of determination (R^2^) and Akaike information criterion (AIC) were used to select the model with the best fit.

### 2.8. Bacterial Strain and Culture Conditions

The reference strain *Staphylococcus aureus* ATCC 25923 (American Type Culture Collection, Manassas, VA, USA) was used for all microbiological assays. Bacterial stocks were maintained at −80 °C and routinely cultured in TSI at 37 °C under aerobic conditions.

### 2.9. Antibacterial Activity Assay

To evaluate the antibacterial activity of NLC, NLC-RIF, and free rifampicin, a fresh bacterial suspension was prepared on Falcon^®^ 5 mL Round Bottom Polystyrene Test Tube at a turbidity equivalent to 0.5 McFarland standard, using a DensiCheck Plus turbidimeter (BioMérieux, Marcy-l’Étoile, France). An inoculum corresponding to 1.5 × 10^7^ colony-forming units (CFUs) was dispensed into each well of a sterile 96-well microplate, followed by the addition of treatment formulations at final concentrations ranging from 0.5 ng/mL to 1 µg/mL in a total volume of 200 µL of TSI per well.

Plates were incubated for 18 h at 37 °C under normoxic conditions. Bacterial growth was quantified by measuring absorbance at 600 nm using a microplate reader (Multiskan Sky, Thermo Scientific, Waltham, MA, USA). The percentage of growth inhibition relative to untreated controls was calculated for each concentration, and dose–response curves were fitted to determine the half-maximal inhibitory concentration (IC_50_) values. All experiments were performed independently at least three times, each in biological quintuplicates.

### 2.10. Metabolic Activity Assay

To evaluate the biosafety of rifampicin, a human hepatocellular carcinoma cell line (HepG2, ATCC^®^ HB-806), kindly provided by Dr. Carlos F. Lagos, was used to assess cell viability. Cells were cultured under standard conditions (37 °C, 5% CO_2_, humidified atmosphere) in DMEM high-glucose supplemented with 10% fetal FBS and penicillin/streptomycin solution. When cultures reached approximately 90% confluence, cells were washed twice with 1X PBS (0.0067 M) and detached by incubation with 0.25% trypsin-EDTA solution at 300× *g* for 5 min. Subsequently, cells were seeded at a density of 1 × 10^4^ cells per well in 96-well plates. Treatments were applied, including NLC, NLC-RIF, and free rifampicin, at increasing concentrations ranging from 0.5 ng/mL to 1 µg/mL.

After 24 h of incubation, cell viability was assessed using the MTT assay at a final concentration of 0.5 mg/mL. Following 1.5 h of incubation at 37 °C, the resulting formazan crystals were dissolved in DMSO, and absorbance was measured at 570 nm using a SYNERGY H1 microplate reader (BioTek Inc., Winooski, VT, USA) with Gen5 v3.16 software. Statistical analysis was performed using two-way ANOVA with multiple comparisons in GraphPad Prism (10.0), and differences were considered statistically significant at *p* < 0.05.

### 2.11. Data Analysis and Statistics

The experiments were performed at least in triplicate, and the data were described as the mean value ± standard deviation (SD). The software used for statistical data analysis was GraphPad Prism software version 10.4.2. Statistical analysis was performed using two-way ANOVA with multiple comparisons and differences were considered statistically significant at *p* < 0.05. In the case of kinetic fit, the DDSolver add-Inn Microsoft Excel program was used.

## 3. Results and Discussion

### 3.1. NLC Physicochemical Properties

In this study, the biological evaluation of rifampicin-loaded nanostructured lipid carriers (NLC-RIF) was conducted, focusing on their antimicrobial activity against *Staphylococcus aureus* and cytotoxicity in HepG2 cells. Physicochemical characterizations were performed to evidence the suitable characteristics of the nanocarrier and its colloidal stability, alongside an assessment of release kinetics, to support the biological findings. NLC and NLC-RIF were successfully prepared by a low-energy hot-emulsion method. The results show that both nanomaterials exhibit nanometric mean hydrodynamic diameter (Table 1), and low polydispersity indices, indicative of a highly monodisperse distribution. Antibiotic loading produced a modest increase in size, yet both systems remained well below 250 nm. It is well established that nanoparticles smaller than ~280 nm can more readily navigate biological barriers—such as mucosal layers and tight endothelial junctions—to access infection sites [33]. However, achieving effective therapeutic delivery requires more than an optimal size; colloidal stability, surface charge, and the ability to protect the cargo until reaching the target are equally critical determinants of in vivo performance [34]. In this work, the low-energy fabrication yielded particles with hydrodynamic diameters below 130 nm and zeta potentials around −4 to −6 mV, balancing steric and electrostatic stabilization without compromising biocompatibility. Moreover, these dimensions compare favorably with other rifampicin-loaded lipid carriers, which often exceed 200 nm in diameter and may face clearance or penetration limitations [35]. This scalable, energy-efficient approach therefore produces NLCs ideally sized for systemic administration and targeted antibiotic release.

On the other hand, NLCs exhibited near-neutral zeta potentials, which alone could compromise colloidal stability by reducing electrostatic repulsion and promoting particle aggregation. However, Gelucire^®^ 44/14 and Tween^®^ 80 in the lipid matrix provide robust steric stabilization: the polyethylene glycol moieties of Gelucire^®^ 44/14 coat the particle surface, while Tween^®^ 80 lowers interfacial tension, together preventing agglomeration. Upon rifampicin loading, the zeta potential became slightly less negative, a change attributable to the drug’s protonated aliphatic amine groups at pH 5.5, which partially neutralized the surface charge (pKa 7.9). Consequently, despite low electrostatic zeta values, the dual steric–electrostatic stabilization mechanism maintains the colloidal integrity of both NLC and NLC-RIF.

According to Guo and colleagues, one of the main limitations in the application of nanomedicine is the lack of pure and well-characterized nanoparticles. In this regard, accurate population size determination is known to be relevant and crucial for drug delivery systems [36]. Nanoparticle tracking analysis (NTA) measurements revealed that empty NLC and rifampicin-loaded NLC (NLC-RIF) differ markedly in both mean size and distribution width. Empty NLC exhibited a mean hydrodynamic diameter of 84.3 ± 1.1 nm, a mode of 76.5 ± 1.0 nm, and a standard deviation of 21.4 ± 3.0 nm. Its D10, D50, and D90 values indicated a relatively narrow, monomodal distribution (Table 2). Upon incorporation of rifampicin, the mean diameter increased to 110.2 ± 3.9 nm with a mode of 89.1 ± 5.5 nm and a larger standard deviation of 33.9 ± 2.8 nm. Correspondingly, D10, D50, and D90 all shifted toward higher values, and the D90–D10 span grew from ~32 nm to ~84 nm, reflecting a broader distribution and a pronounced tail of larger particles. These findings indicate that rifampicin loading leads to particle enlargement and increased polydispersity, likely due to interfacial localization of the drug and reorganization of the lipid–surfactant shell.

**Figure 1:**
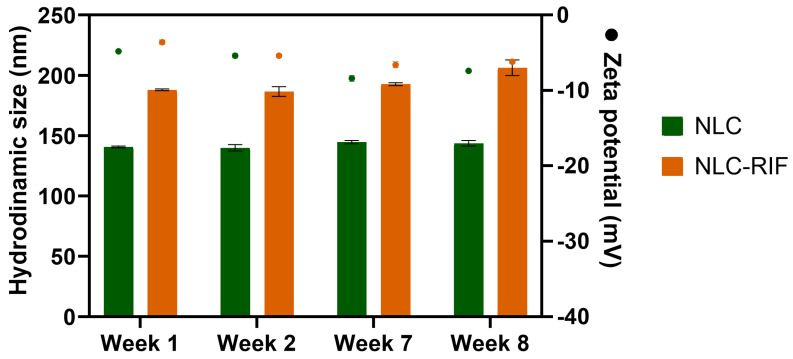
Colloidal stability of NLC and NLC-RIF at 4 °C assessed by hydrodynamic diameter and zeta potential (mean ± SD). n = 3.

On the other hand, the colloidal stability studies demonstrate that the hydrodynamic size and ζ-potential persist without significant changes during at least eight weeks at 4 °C (Figure 1). Blank NLC maintained a mean diameter of 140–145 nm throughout the study (140 ± 2 nm at week 1 vs. 144 ± 2 nm at week 8), whereas NLC-RIF exhibited a modest increase from 185 ± 3 nm to 205 ± 5 nm over the same period. ζ-Potential values for blank NLC remained consistently around −11.5 ± 0.5 mV at week 1 and −12.0 ± 0.6 mV at week 8, indicative of stable electrostatic repulsion, while NLC-RIF showed a slightly less negative surface charge, consistent with partial neutralization by rifampicin at the lipid–water interface. The polydispersity index of both formulations remained below 0.2 throughout the entire eight-week evaluation at 4 °C, demonstrating a consistently narrow size distribution. These results indicate that both NLC and NLC-RIF maintain satisfactory colloidal stability under refrigerated storage.

**Figure 2:**
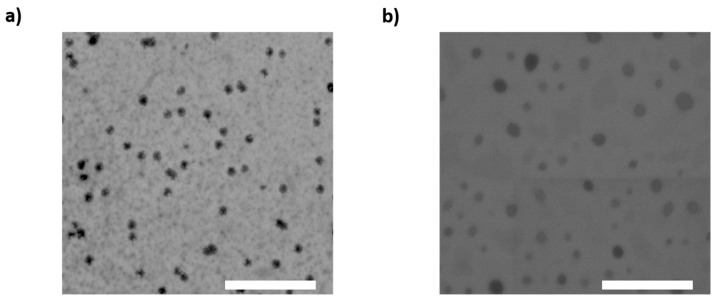
Transmission electron microscopy (TEM) micrograph of (**a**) NLC and (**b**) NLC-RIF. Scale bar: 2 µm.

To investigate the shape and morphology of NLC and NLC-RIF, transmission electron microscopy (TEM) was employed. Both samples exhibited a spherical shape with a monodisperse size distribution, demonstrating the reproducibility of the preparation process (Figure 2). A slight discrepancy was observed between the sizes measured by dynamic light scattering (DLS) and TEM. This difference arises because TEM images reflect the size of the nanoparticles in their solid state after drying, which can lead to a flattened shape and slight deformation at the matrix edges.

### 3.2. Incorporation Efficiency and Drug Loading

Rifampicin is a molecule with pH-dependent low water solubility, reported as 0.31, 0.87, and 1.4 mg/mL at pH 4.0, 7.0, and 9.0, respectively [37]. Additionally, rifampicin exhibits a high log *p* value, which explains its high lipophilicity. However, it contains functional groups such as -OH groups (phenols), an -NH group, and ketone/quinone functions, which give it a certain capacity to form hydrogen bonds, so it is not completely insoluble in water. Therefore, it can behave as an amphipathic molecule in certain environments, which is useful for its incorporation into systems such as lipid nanoparticles (NLC, SLN, liposomes, etc.).

In this case, rifampicin was readily incorporated into the NLCs without complications, yielding an encapsulation efficiency of 98.1 ± 0.6% and a drug loading of 4.5 ± 0.3%. These results indicate that 59.6 µmol of rifampicin is encapsulated, ensuring that 1.64 mg of rifampicin can be available per 1 mL of formulation, independent of the pH of the medium. Consequently, nearly all of the rifampicin introduced during formulation became encapsulated, eliminating the need for organic solvents to enhance loading. While some reports employ organic solvents to improve rifampicin incorporation [38,39], such strategies necessitate additional solvent-removal steps that may leave residual traces and adversely affect in vitro or in vivo performance. In contrast, our method produced a solvent-free NLC-RIF formulation with high encapsulation efficiency.

Regarding the location of drugs within such nanostructures, the literature highlights several key considerations. Firstly, a correlation has been reported between the proportion of the liquid lipid used in the formulation and the drug’s location within the matrix. Specifically, it has been demonstrated that when less than 10% liquid lipid is employed, it forms a distinct drug-loaded phase located at the edges of the nanostructure matrix [12]. Secondly, the emulsification and cooling temperatures significantly affect the initial drug incorporation and its retention within the matrix during storage. During emulsification, typically conducted at approximately 85 °C, rifampicin’s aqueous solubility may slightly increase. As the formulation cools, rifampicin rapidly loses aqueous solubility, leading to enhanced interactions with the lipid phase and subsequent entrapment within the lipid matrix [40]. Additionally, the literature indicates that the cooling process can influence the nanostructure’s conformation. In this context, slow cooling rates (<1–2 °C/min) promote the formation of a β conformation, resulting in a more stable nanostructure and reduced likelihood of drug expulsion from the matrix [41]. Therefore, in this case, rifampicin is expected to be predominantly dispersed within the liquid lipid at the edges of the matrix, which should exhibit a β conformation.

### 3.3. Drug Release and Kinetic Fitting

**Figure 3:**
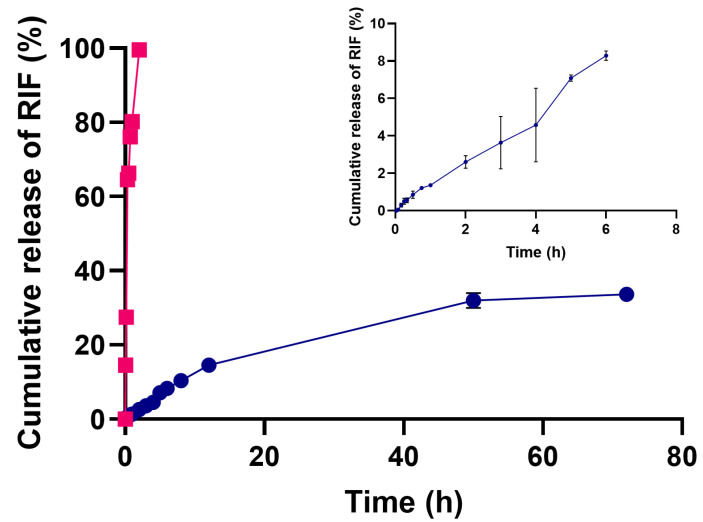
Cumulative release of free rifampicin (magenta line) and rifampicin-loaded NLC (blue line) at 37 °C and pH 7.4 over 72 h (mean ± SD). Inset: release of rifampicin-loaded NLC up to 6 h. n = 3.

The release of rifampicin loaded in NLC and free rifampicin at pH 7.4 and 37 °C is presented in Figure 3. Free rifampicin achieves complete diffusion in approximately 2 h. In contrast, rifampicin release from NLC reaches about 35% within 72 h, corresponding to approximately 1.94 mg of rifampicin. The results show that there is no abrupt release of rifampicin; this is attributed to the drug being incorporated into the lipid matrix and remaining there. This responds to the conformation of an NLC since by containing a liquid lipid, it forms an imperfect matrix that allows for a less structured conformation inside, thus preventing the abrupt expulsion of the drug from the interior of the matrix [12]. In this case, the type of NLC corresponds to type I, since they contain mono-, di-, and triglycerides from Gelucire^®^ 44/14, which generate multiple spaces providing a more suitable environment for the incorporation of the drug [16]. In general, there is little background in the literature on rifampicin loaded into lipid systems, so it is difficult to find direct comparisons; however, there are some precedents. For example, the literature shows that rifampicin loaded in a solid lipid nanoparticle (SLN) presents a burst release, possibly because the nanosystem design only includes solid lipids, therefore the internal conformation is completely compacted and rapidly expels drug molecules to the outside [42]. In another case, in the work conducted by Aliyazdi, et al., they fabricated lipid nanocapsules loaded with rifampicin; and release studies showed a release similar to that obtained in our study, but in a shorter period of time [43]. Meanwhile, the release of rifampicin from polyelectrolyte nanoparticles has been evaluated and in that case, it reaches about 60% at pH 7.4 [19]. Rifampicin is an oxygen-sensitive molecule; therefore, various articles have been described in the literature that use an antioxidant during release tests. Additionally, it has been shown that rifampicin undergoes oxidation reactions usually at neutral or basic pH more rapidly than in acidic media. However, it has been shown that there is no significant difference in the antimicrobial activity of free rifampicin and its oxidized form [44].

The kinetic release data for NLC-RIF were fitted to zero-order, first-order, and Korsmeyer–Peppas models Table 3. The results show that the Korsmeyer–Peppas model provided the best overall description of the release profile (k_KP_ = 4.07; n = 0.99; R^2^ = 0.986; AIC = 56.6), as indicated by its markedly lower AIC value compared with the other models. The diffusional exponent (n = 0.99) corresponds to case II transport, consistent with a release mechanism governed primarily by matrix relaxation and/or erosion rather than simple Fickian diffusion. These results demonstrate that rifampicin release from the NLCs follows an ostensibly zero-order regime in its principal phase, controlled by lipid matrix dynamics.

### 3.4. Antimicrobial Activity

**Figure 4:**
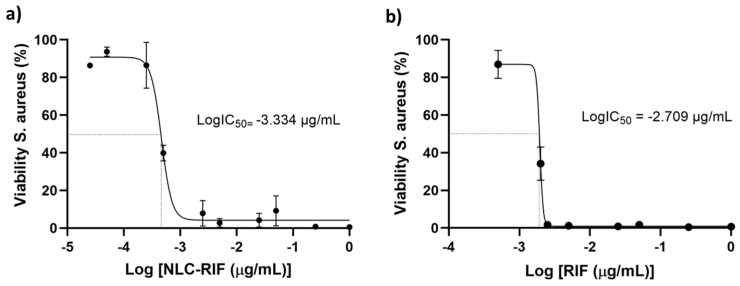
Viability study of *Staphylococcus aureus* at concentrations of 1.0 to 0.0005 µg/mL of rifampicin. (**a**) NLC-RIF and (**b**) RIF. n = 5.

The antimicrobial activity of NLC-RIF against *Staphylococcus aureus* (*S. aureus*) was evaluated through a non-linear regression analysis using a four-parameter logistic (4-PL) model without constraints (Figure 4a). The fitted curve demonstrated a high goodness of fit, with an R^2^ value of 0.9791. The IC_50_ value obtained was 0.0004638 µg/mL, corresponding to a LogIC_50_ of −3.334. The Hill slope was −4.730, indicating inhibitory activity with moderate cooperativity and a relatively steep response curve. The maximum viability observed (Top) was 90.81%, while the minimum viability achieved (Bottom) was 4.215%, resulting in a span of 86.59%. Confidence intervals (95% CI) were narrow for most parameters, supporting the robustness of the model. Altogether, these results suggest that the NLC-RIF nanosystem exhibits potent and efficient antibacterial activity against *S. aureus*, achieving substantial bacterial inhibition at very low concentrations. On the other hand, rifampicin shows a fit yielded an IC_50_ of 0.0019 µg/mL with a Hill coefficient of −19.06, suggesting extremely rapid and cooperative inhibition, characterized by an almost abrupt response curve (Figure 4b). In addition to its markedly lower IC_50_ and steeper Hill slope, the NLC-RIF formulation exhibits a substantially narrower dynamic range than free rifampicin: the transition from ~80% to ~20% bacterial viability occurs over only ~0.2 log µg/mL for NLC-RIF, versus ~0.4 log µg/mL for the unencapsulated drug. This narrow window underscores a pronounced threshold-type response induced by lipid encapsulation, which may help minimize the gap between subtherapeutic and potentially toxic doses allowing it to be useful for applications where effective inhibition is desired but with some modulation. Moreover, both curves display small standard errors at their upper and lower plateaus, attesting to the high reproducibility of the assay, and both NLC-RIF and free rifampicin achieve virtually complete bactericidal activity (≤5 % viability) at concentrations ≥ 10^−2^ mg/mL. These findings reinforce that NLC encapsulation not only enhances potency but also enhances dose–response control and experimental consistency.

Although a complete inhibition of bacterial growth (0% viability) was not achieved under the experimental conditions, the concentration corresponding to the minimal observed bacterial viability (~4%) was estimated at 1.0 µg/mL, suggesting a near-MIC behavior for NLC-RIF nanosystems.

This outcome may be attributed to two factors: intrinsic rifampicin resistance in *S. aureus* and/or the ability of NLCs to overcome this resistance, thereby enhancing antimicrobial efficacy. Rifampicin has been shown to inhibit transcription by interfering with the beta subunit of RNA polymerase; therefore, the mechanism of rifampin resistance in *S. aureus* is determined by mutations in the RpoB gene, which encodes the B subunit of RNA polymerase [45]. The most common mutations are those that cause changes in the amino acid sequence of the RpoB protein [46]. In terms of the interaction of the nanosystem with the microorganism, this result is consistent with what has been reported in the literature, since Gram-positive bacteria have a thin layer of peptidoglycan with teichoic acid and pores, which allows easy penetration of NLC and cause cell membrane damage and cell death [34,47]. In addition, the increase in antimicrobial activity is attributed to the NLC lipids diffusing inward through the thick peptidoglycan layer causing a disruption in the bacteria and enhancing the effect of the antibiotic [48]. For this reason, a lower concentration of rifampicin is required when loaded into the NLC to achieve the same effect as free rifampicin.

In relation to the data obtained in the literature, the group of Tran in 2018 evaluated non-lamellar lyotropic liquid crystalline nanoparticles loaded with rifampicin and obtained an IC_50_ between 0.01 and 0.03 μg/mL [49]. Therefore, the system proposed by our group requires a lower concentration of loaded rifampicin to decrease the IC_50_ by two orders of magnitude. There are more reports in the literature of rifampicin-loaded nanosystems; however, these are nanoparticles that are tested on strains other than *S. aureus* [50,51], or of a different nature than inorganic nanoparticles [52,53], or to evaluations of intracellular infection [54,55], for different purposes [56,57,58,59,60], so they are not comparable with this research.

### 3.5. Cytotoxicity Outcomes

**Figure 5:**
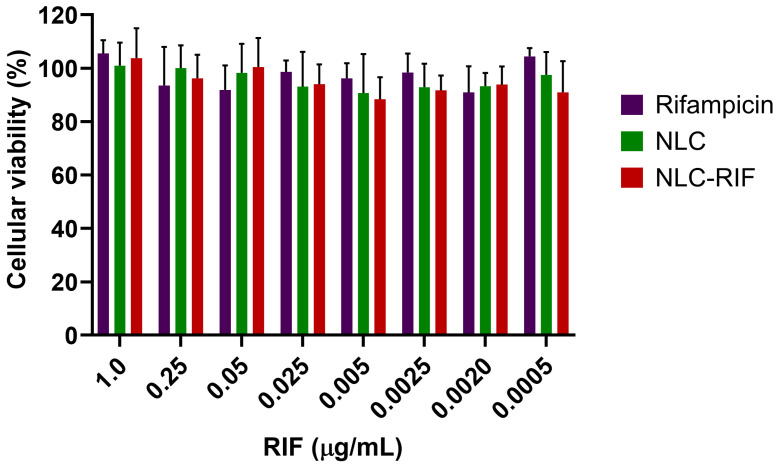
Metabolic effect of rifampicin and NLC formulations treatment on HepG2 cells. Cell viability of HepG2 cells after 24 h of exposure to increasing concentrations of rifampicin (RIF), blank nanostructured lipid carriers (NLCs), and rifampicin-loaded NLC (NLC-RIF). Cells were treated with concentrations ranging from 0.5 ng/mL to 1 µg/mL. Cell viability was assessed using the MTT assay and expressed as a percentage relative to untreated controls. No statistically significant differences were observed among treatments at any concentration tested. Data are presented as mean ± standard deviation (n = 4).

To assess the potential cytotoxicity of the NLC-RIF formulation, a cell viability assay was performed using HepG2 cells exposed to various concentrations of free rifampicin, blank NLCs, and NLC-RIF in a range of 1 to 0.0005 µg/mL, expressed as rifampicin concentration (Figure 5). The results showed that cell viability remained above 90% under all tested conditions, with no significant differences observed between treatments. Neither free rifampicin, blank NLCs, nor NLC-RIF negatively affected the viability of HepG2 cells, even at the highest tested concentration (1 µg/mL), indicating good cellular tolerability of the nanostructured formulation.

The excellent cell viability observed in HepG2 cells following exposure to NLC-RIF indicates that the formulation does not exert significant cytotoxic effects at concentrations up to 1 µg/mL. This result is consistent with the NLC formulation, as Gelucire^®^ 44/14 has been recognized as GRAS (generally recognized as safe) [61]; Miglyol^®^ 812 has been studied as a safe excipient [62,63]. In the case of Tween^®^ 80, its surfactant properties can disrupt cell membranes [64,65]; however, this material interacts with the NLC surface, and at its present concentration, it is not sufficient to cause destabilization and subsequent cell death.

The comparable viability values across free rifampicin, blank NLCs, and NLC-RIF suggest that encapsulation of rifampicin within the lipid matrix does not introduce additional cytotoxicity. These findings support the suitability of NLC-RIF for further development as a safe delivery system, particularly for therapeutic applications requiring systemic exposure or long-term treatment.

A study evaluating an NLC against HepG2 cells is reported in the literature; however, the objective of this study was to investigate an anticancer NLC. Despite this, based on the published results, it can be concluded that NLC cytotoxicity is not predominant [66]. Gundogdu and colleagues developed an NLC with two types of Gelucire^®^, oleic acid, compritol, Span^®^ 80, Tween^®^ 80, and lipoid derivatives; therefore, the materials are similar to those used by our group. In this case, the nanosystem was studied in a gastric adenocarcinoma cell line. Although the authors do not show the cytotoxicity of NLC, all nanocarriers showed cell viability above 80% [67]. On the other hand, the Chandan group manufactured an NLC composed of glyceryl monostearate, castor oil, and Pluronic F68. The authors evaluated cytotoxicity using MTT in Caco-2 cells and determined that the unloaded NLCs exhibited cell viability greater than 80% at all concentrations tested [68].

## 4. Conclusions

The development of nanostructured lipid carriers loaded with rifampicin resulted in a stable and biocompatible nanosystem with enhanced antibacterial activity against *Staphylococcus aureus*. The NLC-RIF formulation exhibited favorable physicochemical characteristics, sustained drug release, and a significantly lower IC_50_ compared to free rifampicin, indicating improved potency. In addition, the system maintained excellent colloidal stability over extended storage and demonstrated high cell viability in hepatic cells, supporting its safety for biomedical use. These findings position NLC-RIF as a promising platform for targeted antibiotic delivery. Future work should explore the in vivo pharmacokinetics, biodistribution, and therapeutic efficacy of this nanosystem, as well as its applicability against other clinically relevant bacterial strains and biofilm-associated infections.

## Figures and Tables

**Table 1:** Characterization of NLC and NLC-RIF through hydrodynamic diameter (nm ± SD), PdI (±SD), and zeta potential (mV ± SD) by dynamic light scattering (DLS) and particle concentration by nanoparticle tracking analysis (NTA).

Nanomaterial	Hydrodynamic Size ± SD (nm)	Polydispersity Index ± SD	Zeta Potential ± SD (mV)	Particle Concentration ± SD (Particle/mL)
NLC	98.6 ± 2.2	0.06 ± 0.03	−4.9 ± 0.1	2.1 × 10^17^ ± 1.8 × 10^16^
NLC-RIF	121.5 ± 2.0	0.18 ± 0.03	−3.4 ± 1.6	8.7 × 10^16^ ± 1.2 × 10^15^

**Table 2:** Analysis of NLC and NLC-RIF size distributions using NTA.

Nanosystem	Nanoparticle Tracking Analysis (nm ± SD)		
	D10	D50	D90
NLC	84.3 ± 1.1	80.0 ± 0.2	100.4 ± 2.6
NLC-RIF	75.3 ± 2.2	101.7 ± 4.6	159.1 ± 8.9

**Table 3:** Kinetic fit evaluation to NLC-RIF at 37 °C. In equations, Q_0_ and Q_t_ are the initial amount of rifampicin and the amount of rifampicin dissolved at time t, respectively. Q_t_/Q_∞_ is the fractional release of rifampicin, K_0_ is the zero-order constant, K_1_ is the first-order constant, k_KP_ is the Korsmeyer–Peppas constant and n is the diffusional exponent.

	Zero-Order			First Order			Korsmeyer–Peppas			
	k_0_ (%h^−1^)	R^2^	AIC	k_1_ (h^−1^)	R^2^	AIC	k_KP_	*n*	R^2^	AIC
NLC-RIF	1.64	0.879	130.2	0.04	0.995	75.3	4.07	0.99	0.986	56.6

## Data Availability

The manuscript contains the reported data. Additional relevant data can be obtained upon request from the corresponding author.
